# The Enterohemorrhagic Escherichia coli Effector EspW Triggers Actin Remodeling in a Rac1-Dependent Manner

**DOI:** 10.1128/IAI.00244-17

**Published:** 2017-08-18

**Authors:** Pamela Sandu, Valerie F. Crepin, Hauke Drechsler, Andrew D. McAinsh, Gad Frankel, Cedric N. Berger

**Affiliations:** a MRC Centre for Molecular Bacteriology and Infection, Department of Life Sciences, Imperial College London, London, United Kingdom; b Centre for Mechanochemical Cell Biology, Division of Biomedical Cell Biology, Warwick Medical School, University of Warwick, Coventry, United Kingdom; University of Texas at Austin

**Keywords:** EHEC, EPEC, Kif15, Rac1, Rho GTPase, actin, *espW*

## Abstract

Enterohemorrhagic Escherichia coli (EHEC) is a diarrheagenic pathogen that colonizes the gut mucosa and induces attaching-and-effacing lesions. EHEC employs a type III secretion system (T3SS) to translocate 50 effector proteins that hijack and manipulate host cell signaling pathways, which allow bacterial colonization and subversion of immune responses and disease progression. The aim of this study was to characterize the T3SS effector EspW. We found *espW* in the sequenced O157:H7 and non-O157 EHEC strains as well as in Shigella boydii. Furthermore, a truncated version of EspW, containing the first 206 residues, is present in EPEC strains belonging to serotype O55:H7. Screening a collection of clinical EPEC isolates revealed that *espW* is present in 52% of the tested strains. We report that EspW modulates actin dynamics in a Rac1-dependent manner. Ectopic expression of EspW results in formation of unique membrane protrusions. Infection of Swiss cells with an EHEC *espW* deletion mutant induces a cell shrinkage phenotype that could be rescued by Rac1 activation via expression of the bacterial guanine nucleotide exchange factor, EspT. Furthermore, using a yeast two-hybrid screen, we identified the motor protein Kif15 as a potential interacting partner of EspW. Kif15 and EspW colocalized in cotransfected cells, while ectopically expressed Kif15 localized to the actin pedestals following EHEC infection. The data suggest that Kif15 recruits EspW to the site of bacterial attachment, which in turn activates Rac1, resulting in modifications of the actin cytoskeleton that are essential to maintain cell shape during infection.

## INTRODUCTION

The human pathogens enterohemorrhagic Escherichia coli (EHEC) and enteropathogenic E. coli (EPEC) (1) and the mouse pathogen Citrobacter rodentium (CR) (2) constitute a bacterial family that colonizes the intestinal mucosa and induces the formation of attaching-and-effacing (A/E) lesions. The A/E lesions are characterized by effacement of the brush border microvilli, intimate attachment of the bacteria to the apical membrane of host epithelial cells, and induction of actin polymerization beneath the attached bacteria (3). EPEC, EHEC, and C. rodentium employ a filamentous type III secretion system (T3SS) (4), located within the locus of enterocyte effacement (LEE) (5), to translocate a plethora of effector proteins directly from the bacterial cell into host cell cytoplasm (6). Of the translocated effectors, five (Tir, EspZ, EspH, EspG, and Map) are LEE encoded. The effector Tir plays a key role in formation of A/E lesions *in vivo* (7) and in actin-rich pedestals in cultured cells (8). Following clustering by the LEE-encoded outer membrane adhesin intimin, EPEC Tir (Tir_EPEC_) and C. rodentium Tir (Tir_CR_) bind Nck, while EHEC Tir (Tir_EHEC_) binds the adaptor proteins IRTKS and/or IRSp53 (9, 10) and recruits the effector TccP/EspFu (11, 12). The Tir signaling pathways then converge on N-WASP and the ARP2/3 complex, leading to actin polymerization (13).

The actin cytoskeleton, which is targeted by many bacterial pathogens, is essential for cell integrity, motility, membrane trafficking, and shape changes (14). Rho GTPases, which belong to the family of Ras-related small GTPases, are key regulators of various cellular processes, including actin polymerization, microtubule dynamics, vesicle trafficking, cell polarity, and cytokinesis (15). The best-characterized members of the Rho GTPase family are RhoA, Rac1, and Cdc42, the activation of which leads to the assembly of stress fibers, lamellipodia/ruffles, and filopodia, respectively (16). Switching of Rho GTPases from an inactive GDP-bound state to an active GTP-bound state is mediated by guanine nucleotide exchange factors (GEFs). The switch back from the active GTP to an inactive GDP-bound state is regulated by GTPase-activating proteins (GAPs). In their GTP-bound conformation, Rho GTPases interact with and activate downstream target effectors, such as serine/threonine kinases, tyrosine kinases, lipid kinases, lipases, oxidases, and scaffold proteins (17). As Rho GTPases are important regulators of the actin cytoskeleton, bacterial pathogens have evolved strategies to subvert their signaling during infection.

Bacterial guanine nucleotide exchange factors, which belong to the SopE family, act as bacterial Rho GEFs to activate the host Rho GTPase (18). The A/E pathogen effector Map induces filopodia via Cdc42 at the site of attachment (19, 20), EspM promotes stress fibers via RhoA activation (21), and EspT triggers ruffle and lamellipodia formation by Rac1 (22). A/E pathogens also translocate effectors that inactivate Rho GTPases. EspH globally inactivates DH-PH domain mammalian Rho-GEFs but not the bacterial Rho-GEFs (23). Tir antagonizes the activity of Map as it downregulates formation of filopodia (24), while EspO2 interacts with EspM2 and blocks formation of the stress fibers (25).

Using a transfection-based screen, we recently identified EspW_EHEC_ as a regulator of actin filament organization. EspW has been shown previously to be secreted by EHEC and translocated into mammalian cells in a type 3-dependent manner (26). However, until now, no function has been identified for this effector. The aim of this study was to investigate the role of EspW during EHEC infection and its putative role as a Rho GTPase regulator.

## RESULTS

### Screening of *espW* in EPEC clinical isolates.

EspW is a 352-amino-acid effector and is located in the SP17 pathogenic island, which also encodes EspM2 and members of the NleG family (see Fig. S1A in the supplemental material). So far, EspW has been reported only in EHEC O157:H7 and EPEC B171 (O111:H^−^) strains, with no homologs among other bacterial species. Using the BLAST algorithm with EspW as the index protein, we confirmed that it was present in the sequenced EHEC O157:H7 strains, in five non-O157:H7 EHEC strains (O111:H^−^, O111:H11, O26:H11, O103:H2, and O103:H25), and in Shigella boydii (Fig. S1B). Furthermore, a putative coding sequence for a truncated version of EspW containing the N-terminal 206 amino acids (EspW_1–206_) was present in two EPEC strains (CB9615 and RM12579) belonging to serotype O55:H7 (Fig. S2), a progenitor of EHEC O157:H7 (27). In order to determine if either the long or short versions of *espW* are present in other EPEC strains, we screened by PCR a collection of 132 clinical isolates available in our laboratory. This revealed that the long version of *espW* is present in 52% of the tested stains (Table 1). Furthermore, *espW_1–206_* was found in 10 of the 132 (8%) strains tested (Table 1). Interestingly, 9 of the 10 *espW_1–206_* genes belonged to serotype O55:H7. Neither of the *espW* variants was found in C. rodentium and the prototype EPEC strain E2348/69, while the prototype atypical EPEC strain E110019 (O111:H9) contains the long version of *espW*.

**TABLE 1:** Distribution of *espW* and *espW_1–206_* among 132 clinical EPEC isolates^*a*^

Serogroup (no. of strains)	No. of strains carrying *espW*	Serotype (no. of strains/total no. of strains tested)
ONT (3)	3	H7 (1/1); H45 (1/1); H^−^ (1/1)
O13 (1)	1	H^−^ (1/1)
O26 (13)	9	H^−^ (4/8); H11 (5/5)
O49 (1)	1	H^−^ (1/1)
O55 (24)	10	H^−^ (3/11); H6 (5/5); H7 (1/5); H34 (1/3)
O86 (5)	3	H8 (0/2); H34 (3/3)
O104 (1)	1	H2 (1/1)
O109 (1)	1	H9 (1/1)
O111 (12)	5	H^−^ (2/4); H2 (3/3); H9 (0/1); H12 (0/1); H21 (0/1); H25 (0/2)
O114 (3)	2	H^−^ (0/1); H2 (2/2)
O119 (29)	14	H2 (4/11); H4 (0/1); H6 (10/17)
O123 (1)	1	H^−^ (1/1)
O125 (3)	1	H6 (1/3)
O126 (4)	1	H^−^ (1/1); H27 (0/3)
O127 (8)	3	H^−^ (1/1); H6 (2/3); H27 (0/1); H40 (0/3)
O128 (6)	4	H^−^ (0/1); H2 (4/4); H35 (0/1)
O142 (7)	5	H6 (3/3); H34 (2/4)
O153 (1)	1	H^−^ (1/1)
O154 (1)	1	H9 (1/1)
O177 (1)	1	H11 (1/1)

a *espW* was present in 68 out of 132 EPEC strains screened; 10 out of the 64 PCR-negative strains (O26:H^−^ [1 strain], O55:H^−^ [5 strains], and O55:H7 [4 strains]) were *espW_1–206_* positive. The following strains were *espW* and *espW_1–206_* negative: O2:H49 (1 strain), O6:H19 (2 strains), O45:H^−^ (1 strain), O85:H^−^ (1 strain), and O118:H5 (2 strains).

### EspW interacts with the C terminus of Kif15.

**FIG 1:**
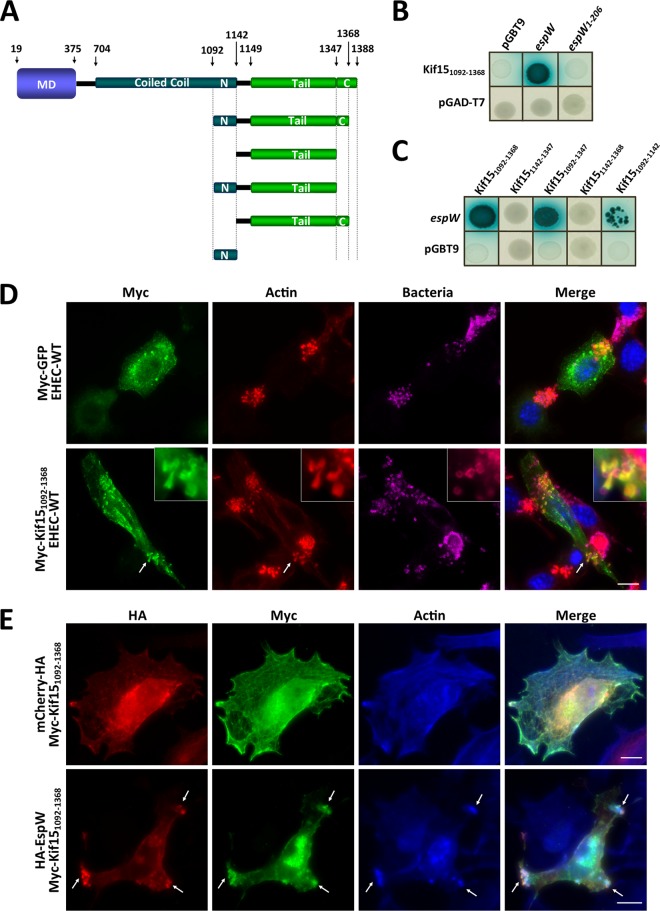
Kif15 interacts with EspW. (A) Schematic representation of Kif15. (B) Direct yeast two-hybrid assay revealed that EspW, but not EspW_1–206_, interacts with Kif15_1092–1368_. (C) EspW interacts with Kif15_1092–1368_, Kif15_1092–1347_, and Kif15_1092–1142_, but not Kif15_1142–1368_ and Kif15_1142–1347_, by direct yeast two-hybrid assay. (D) Following infection of transfected Kif15_1092–1368_ (green) cells, Kif15_1092–1368_ localized at the actin (red) pedestals (white arrows), under adherent EHEC (magenta). DNA was visualized by Hoechst staining (blue). (E) Ectopically expressed Kif15_1092–1368_ (green) colocalized with EspW (red) and actin (magenta), but not mCherry, in Swiss 3T3 cells. Bar, 10 μm.

In order to identify the EspW host cell partner protein, we performed a yeast two-hybrid screen using a HeLa cell cDNA library as bait and identified the carboxy terminus of Kif15, Kif15_1092–1368_, as a putative partner. The interaction was confirmed by direct yeast two-hybrid (DY2H). Importantly, Kif15_1092–1368_ interacted with the full-length EspW (Fig. 1B) but not with EspW_1–206_. To further map the binding site of EspW to Kif15, five Kif15 truncation fragments were generated and tested by DY2H (Fig. 1A). An empty pGAD-T7 plasmid was used as a negative control. No growth was observed on selected media (QDO) when yeast were cotransformed with EspW and Kif15_1142–1347_, Kif15_1142–1368_, or the negative control. In contrast, growth was seen following cotransformation with EspW and Kif15_1092–1347_ or Kif15_1092–1142_ (Fig. 1C), suggesting that the C-terminus coil-coil domain of Kif15 plays an important role in the interaction with EspW.

### Kif15 localizes to the pedestals and colocalizes with EspW.

We aimed to determine the localization of Kif15 during EHEC infection. However, we were unable to detect endogenous Kif15, and localization of overexpressed Kif15 was difficult to detect due to poor transfection efficiency. Accordingly, we determined the localization of ectopically expressed Kif15_1092–1368_, used in the DY2H, following EHEC infection of transfected Swiss 3T3 cells. Cells expressing myc-green fluorescent protein (GFP) were used as a negative control. Immunofluorescence (IF) microscopy revealed that Kif15_1092–1368_, but not GFP, localized to the actin pedestals at the site of EHEC attachment (Fig. 1D). Interestingly, cells transfected with Kif15_1092–1368_ and infected with an EHEC Δ*espW* strain present a similar recruitment of Kif15_1092–1368_ into the pedestal (Fig. S3), suggesting EspW is not required for localization of Kif15 to the pedestal.

We next aimed to determine if Kif15_1092–1368_ and EspW colocalized. For this, we first tried to hemagglutinin (HA) tag EspW in EHEC; however, no signal could be detected by IF. Therefore, we cotransfected cells with pRK5-HA-*espW* and pRK5-Myc-*kif15*_1092–1368_. pRK5-HA-*mCherry* served as a negative control. EspW and Kif15_1092–1368_ colocalized, whereas no colocalization was observed between mCherry and Kif15_1092–1368_ (Fig. 1E). Interestingly, EspW and Kif15_1092–1368_ were also present at membrane sites showing actin reorganization.

### EspW triggers actin remodelling in a Rac1-dependent manner.

**FIG 2:**
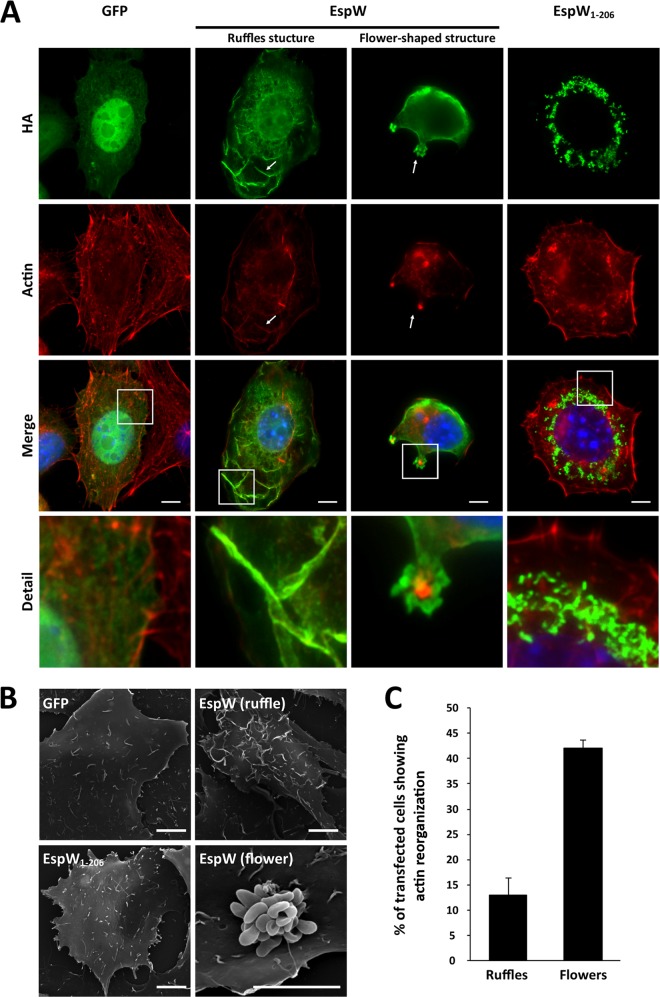
EspW induces actin rearrangement. (A) Ectopic expression of HA-EspW (green) induces either actin (red) ruffles or flower-shaped structures. No actin modification can be observed with HA-EspW_1–206_ or GFP (green). DNA was visualized by Hoechst staining (blue). White arrows indicate colocalization of EspW with actin. Bar, 5 μm. (B) SEM of transfected cells. (C) Quantification of actin structure observed in transfected cells.

In order to determine if EspW is responsible for the observed actin reorganization (Fig. 1E), we transfected cells with pRK5-HA-*espW*, pRK5-HA-*espW_1–206_*, or pRK5 encoding GFP as a negative control. Immunofluorescence staining (Fig. 2A) and scanning electron microscopy (SEM) (Fig. 2B) revealed that the full-length EspW triggered formation of either membrane ruffles (13% of transfected cells) or flower-shaped structures (42% of transfected cells), which were rich in actin and colocalized with EspW (Fig. 2A to C). EspW_1–206_ showed aggregative localization dispersed within the cell with an actin structure similar to those seen in the GFP control cells (Fig. 2A and B).

**FIG 3:**
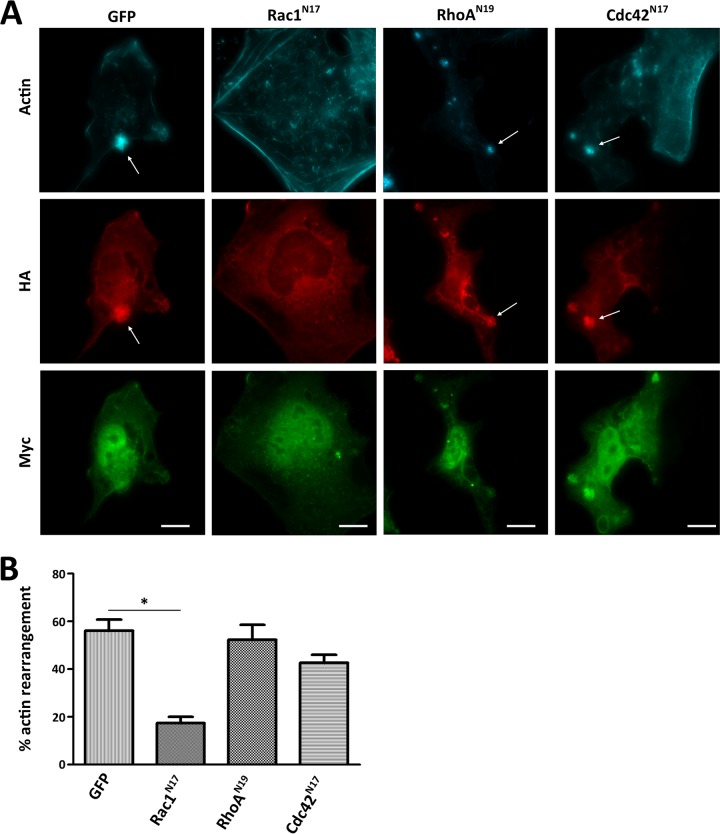
EspW-induced actin reorganization is Rac1 dependent. (A) Cotransfection of HA-EspW (red) with myc-Rac1^N17^ (green), but not with GFP, Myc-Cdc42^N17^, and Myc-RhoA^N19^, inhibited actin (blue) rearrangement (white arrow). Bar, 10 μm. (B) Quantification of cotransfected cells showing actin rearrangement. The percentage was calculated by counting 100 transfected cells (in triplicate) from three independent experiments. Results are presented as means ± SD. *, *P* < 0.05.

In order to determine if EspW-induced actin remodelling requires RhoA, Rac-1, or Cdc42, we cotransfected HeLa cells with pRK5-HA-*espW* and a dominant negative of each of the GTPases (Rac1^N17^, RhoA^N19^, and Cdc42^N17^). The cotransfected cells were assessed by IF for the presence of actin-rich flower-shaped structures. This revealed that inactivation of either RhoA or Cdc42 had no effect on the ability of EspW to induce actin reorganization (Fig. 3A). In contrast, inhibition of Rac1 significantly compromised the ability of EspW to induce actin rearrangements (Fig. 3A and B).

### Deletion of *espW* induces cell shrinkage that could be overcome by Rac1 activation.

**FIG 4:**
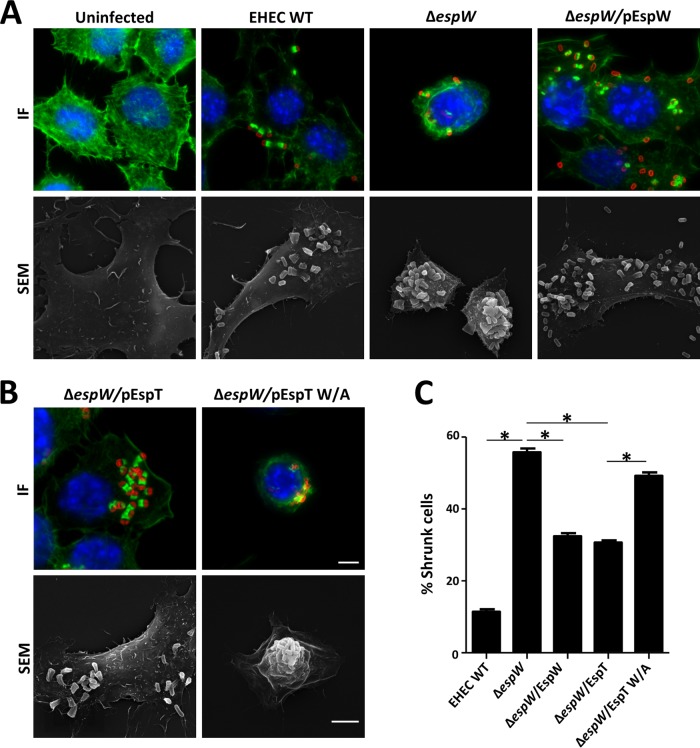
EHEC Δ*espW* mutant induces cell shrinkage. (A) Shrinking of cells (visualized by IF, with actin in green and DNA in blue, and SEM) was observed following infection with an EHEC Δ*espW* strain in comparison to cells left uninfected or infected with WT EHEC (red) or an EHEC Δ*espW* strain complemented with pEspW. Bar, 5 μm. (B) Cells infected with the EHEC Δ*espW* strain expressing EspT_w/A_ shrunk compared to cells infected with the EHEC Δ*espW* strain expressing WT EspT. Bar, 5 μm. (C) Quantification of phenotype observed in panel B (100 infected cells in triplicate) following infection with WT EHEC, an EHEC Δ*espW* strain, and EHEC Δ*espW* strains expressing EspW (Δ*espW*/EspW), EspT (Δ*espW*/EspT), and EspT W/A (Δ*espW*/EspT W/A). *, *P* < 0.05.

To assess the role of EspW during infection, cells were infected for 3 h with wild-type (WT) EHEC, an EHEC Δ*espW* strain, or an EHEC Δ*espW* strain complemented with pEspW. Immunofluorescence reveals that infection with the EHEC Δ*espW* strain induced significant cell shrinkage (56%) compared to infection with WT EHEC (12%) (Fig. 4A). Partial complementation was observed for the cell infected with the EHEC Δ*espW* strain complemented with pEspW (32%) (Fig. 4C).

To determine if the cell shrinkage was linked with lack of activation of Rac1, cells were infected with an EHEC Δ*espW* strain overexpressing EspT, an effector known to activate Rac1 (22). The EspT_W/A_ mutant, lacking the GEF activity of EspT, was used as a negative control (Fig. 4B). Expression of WT EspT significantly reduced cell shrinkage (31%) compared with cells infected with the EHEC Δ*espW* strain complemented with pEspT_W/A_ (50%) (Fig. 4C).

**FIG 5:**
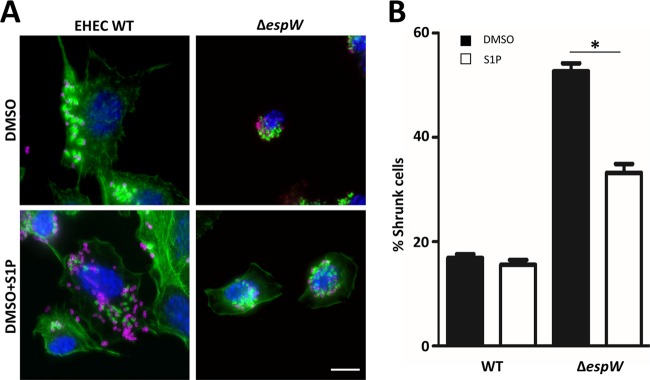
Rac1 activation prevents cell shrinkage. (A) Immunofluorescence microscopy of Swiss cells (visualized with actin in green and DNA in blue) infected (magenta) with the WT or Δ*espW* EHEC strain in the presence or absence of 100 nM S1P. The presence of S1P prevented cell shrinkage. Bar, 10 μm. (B) Quantification of phenotype observed in panel B (100 cells in triplicate) infected with the WT or Δ*espW* EHEC strain in the presence (white bar) or absence (black bar) of S1P. *, *P* < 0.05.

In order to confirm that the cell shrinkage was caused by the lack of Rac1 activation, we chemically induced activation of Rac1 during infection by adding 100 nM sphingosine 1-phosphate (S1P) to the culture medium (28) and quantified the number of shrunken cells after infection (Fig. 5A and B). S1P treatment significantly reduced shrinking of cells infected with the EHEC Δ*espW* strain from 53% to 33% (Fig. 5B). These results suggest that EspW activates Rac-1, which stabilizes the shape of infected cells.

## DISCUSSION

In this study, we found that *espW* is common among clinical EHEC and EPEC isolates; an *espW* orthologue is also found in Shigella boydii. The majority of the EPEC strains contain the full-length *espW* gene, while others, mainly belonging to EPEC O55:H7, encode a truncated EspW isoform. Although the truncated form of EspW does not induce actin reorganization, it is possible that it has other biological functions.

Using a two-hybrid screen, we identified Kif15 as a specific partner of the full-length EspW isoform. Human Kif15 is a multimeric protein of 1,388 amino acids which belongs to the kinesin family (29). It has an N-terminal motor domain (residues 19 to 375) followed by a long alpha-helical rod-shaped stalk predicted to form an interrupted coiled coil. The C-terminal region has been shown to contain a putative actin interacting region (residues 743 to 1333) (30). Moreover, in HeLa cells, Kif15 has been shown to concentrate on spindle poles and microtubules in early mitosis and to localize with actin in late mitosis (31). One possibility is that Kif15 switches binding from one filament system to the other, while another possibility is that Kif15 associates with the most abundant cytoskeletal filament system (31). In this study, we mapped the EspW binding site to a segment of Kif15, amino acids 1092 to 1142. This segment is a known binding site for both Ki-67 (1017 to 1237) and actin (743 to 1333). The exact role of Kif15 during infection is still unclear, as labeling of EspW in EPEC did not allow us to localize the effector during infection. However, its recruitment to the pedestal during EPEC infection is independent of EspW. We therefore hypothesize that Kif15 recruits EspW and determines its spatial distribution, similar to the function of NHERF1 or NHERF2 toward the effector Map (32).

EPEC and EHEC, like many other enteric pathogens, target actin cytoskeleton as part of their infection strategy. The hallmark of EPEC and EHEC infection of cultured cells is formation of actin pedestal-like structures underneath the attached bacteria. In EPEC, formation of these structures is dependent on the effector Tir and activation of N-WASP and independent of activation of mammalian Rho GTPases (33). However, EspH, which is a global inhibitor of endogenous mammalian GEFs (23), is required for efficient actin pedestal elongation (34), suggesting that Rho GTPases are partially involved in this process. Importantly, EPEC and EHEC translocate several effectors, belonging to the SopE family, which have a GEF activity toward mammalian Rho GTPases (18). *In vitro*, EspT, which activates Rac1, triggers formation of ruffles or lamelipodia, and *in vivo* it induces expression of KC and tumor necrosis factor alpha (TNF-α) (35). In this study, we found that EspW also appears to activate Rac1, either directly or indirectly, in a compartmentalized fashion; this is in contrast to EspT, which has a more global effect. Nonetheless, the phenotype of *espW* deletion could be partially complemented by *espT*, suggesting some activity overlap. Due to poor solubility, we were not able to identify whether EspW directly activates Rac1. Importantly, multiple biological systems revealed that activation or inhibition of the Rho GTPase has to be fine-tuned both spatially and temporally. Their overactivation or inhibition have detrimental effects leading to activation of alarm signals (36) or apoptosis (37). During EPEC infection, activation of Cdc42 is limited to the bacterial binding sites (19), followed by rapid inhibition by Tir (19). The effector EspO, expressed by a selection of EPEC and EHEC strains, has been reported to inactivate EspM2 (RhoA GEF). Interestingly, deletion of *espO1* and *espO2* leads to cell shrinkage in an EspM2-dependent manner (25). Rac1 and RhoA have antagonistic effects (38). Interestingly, we found that cells infected with EHEC expressing EspM1 and EspM2 but missing EspW undergo cell shrinkage. This cell shrinkage phenotype was not associated with decreased cell attachment or with any signs of cell death, including nucleus condensation, loss of membrane permeability, or membrane blebbing, for the duration of the experiment. Interestingly, we found that EPEC and EHEC strains expressing EspM also express either EspT or EspW, suggesting that activation of RhoA and Rac1 need to be coordinated during infection. Furthermore, deletion of Rac1 impairs focal adhesion complex formation and cell spreading (39). Taken together, these observations suggest that EPEC and EHEC have developed a complex mechanism to control cell shape by manipulating the localization and activation of RhoA and Rac1. Any dysregulation leading to an uncontrolled activation leads to dramatic cell morphology changes. Further studies will be needed in order to understand the spatiotemporal regulation of the Rho GTPase during EPEC and EHEC infections.

## MATERIALS AND METHODS

### Bacterial strains, growth conditions, and cell culture.

The bacterial strains used in this study and their origins are listed in Table 2. Bacteria were grown from a single colony in Luria-Bertani (LB) broth in a shaking incubator (200 rpm) at 37°C for 18 h or on agar supplemented with ampicillin (100 μg/ml) or kanamycin (50 μg/ml). For cell infections, EHEC strains were grown in LB in a shaking incubator (200 rpm) at 37°C for 8 h and then subcultured (1/500) in Dulbecco's modified Eagle's medium (DMEM) with low glucose and grown overnight at 37°C without agitation in a 5% CO_2_ incubator (primed culture).

**TABLE 2:** List of strains and plasmids

Strain or plasmid	Description/function	Source/reference
Strains		
85-170	EHEC O157:H7, *stx* mutant	44
ICC1111	85-170 Δ*espW*	This study
AH109	S. cerevisiae MATα mating type with ADE2, HIS3, MEL1, and LacZ reporters for interaction and TRP1 and LEU2 selection markers	Clontech
Y187	S. cerevisiae MATα mating type with MEL1 and LacZ reporters and TRP1 and LEU2 selection markers	Clontech
Plasmids		
pRK5-HA (Amp^r^)	Eukaryotic expression vector of HA-tagged protein	45
pICC1396	pRK5 expressing HA-tagged mCherry	This study
pICC1727	pRK5 expressing HA-tagged EspW	This study
pICC1728	pRK5 expressing HA-tagged EspW_1–206_	This study
pRK5-myc (Amp^r^)	Eukaryotic expression vector of myc-tagged protein	Clontech
pICC563	pRK5 expressing myc-tagged GFP	46
pRK5-myc-Rac1^N17^	pRK5 expressing myc-tagged Rac1^N17^	47
pRK5-myc-RhoA^N19^	pRK5 expressing myc-tagged RhoA^N19^	47
pRK5-myc-Cdc42^N17^	pRK5 expressing myc-tagged Cdc42^N17^	47
pICC1914	pRK5 expressing myc-tagged Kif15_1092–1368_	This study
pSA10 (Amp^r^)	pKK177-3 derivative containing *lac*^I^	42
pICC1732	pSA10 derivative expressing EspW	This study
pICC461	pSA10 derivative expressing EspT	22
pICC1205	pSA10 derivative expressing EspT_W/A_	22
pKD46 (Amp^r^)	Coding for the lambda red recombinase	40
pSB315 (Kan^r^)	Coding for the kanamycin resistance *aphT* cassette	48
TOPO Blunt II (Kan^r^)	TOPO cloning of blunt PCR products	Invitrogen
pGBT9	Gal4 DNA binding domain, selective for −Trp medium expression for proteins in yeast	Clontech
pICC1714	pGBT9 derivative expressing EspW	This study
pICC1715	pGBT9 derivative expressing EspW_1–206_	This study
pGAD-T7-AD	Yeast two-hybrid prey expression vector	Clontech
pICC1723	pGAD derivative expressing Kif15_1092–1368_	This study
pICC1724	pGAD derivative expressing Kif15_1142–1347_	This study
pICC1725	pGAD derivative expressing Kif15_1092–1347_	This study
pICC1726	pGAD derivative expressing Kif15_1142–1368_	This study
pICC1752	pGAD derivative expressing Kif15_1092–1142_	This study

Saccharomyces cerevisiae (AH109) was grown in YPDA medium (20 g/liter Difco peptone, 10 g/liter yeast extract, 2% glucose, and 0.003% adenine hemisulfate) for 48 h at 30°C. For the yeast two-hybrid screen, clones containing interaction partners were selected on high-stringency quadruple-dropout (QDO) medium lacking leucine, tryptophan, histidine, and adenine in the presence of X-α-Gal (Clontech Laboratories, Inc.). Successful transformation with bait and prey plasmids was selected by plating on double-dropout (DDO) medium lacking leucine and tryptophan. Bait-prey interactions were assessed by streaking the transformed clones from DDO onto QDO selection medium.

Swiss 3T3 and HeLa cells were maintained in DMEM with 4,500 mg/ml glucose (Sigma) or DMEM with 1,000 mg/ml glucose (Sigma), respectively, supplemented with 10% (vol/vol) heat-inactivated fetal calf serum (FCS; Gibco), 4 mM GlutaMAX (Gibco), and 0.1 mM nonessential amino acids at 37°C in 5% CO_2_.

### Plasmids and molecular techniques.

Plasmids used in this study are listed in Table 2, and primers are listed in Table S1 in the supplemental material.

The EHEC Δ*espW* (ICC1111) strain was generated using a lambda red-based mutagenesis system (40) in which *espW* was replaced by a kanamycin cassette. Plasmid pSB315 was the source of the kanamycin resistance gene (*aphT*), which was purified following EcoRI restriction digestion. Primer pair P23/P24 was used to PCR amplify *espW* with 500-bp upstream and downstream flanking regions from E. coli O157:H7 (85-170) genomic DNA. The PCR product was cloned into TOPO Blunt II vector (Invitrogen), and *espW* was removed by inverse PCR using the primer pair P25/P26. The linear PCR product was then EcoRI digested to allow ligation of the kanamycin cassette. The insert was then amplified using the primer pair P23/P24 and the PCR product electroporated into WT EHEC containing pKD46 encoding the lambda red recombinase. Transformants were selected on kanamycin plates, and the deletion of *espW* was confirmed by PCR and DNA sequencing (using primer pair P27/P28).

*espW* and *espW_1–206_* were cloned into the bacterial expression vector pRK5-HA following amplification from 85-170 genomic DNA using primer pairs P1/P2 and P1/P3, generating plasmids pICC1727 and pICC1728. *mCherry* was amplified from pmcherry-miniSOG-C1 (41) using primers P4/P5, generating plasmid pICC1396. Plasmid pICC1727 was used as the template to amplify *espW* (P10/P11) and further cloned into pSA10 (42), generating plasmid pICC1732.

*espW* and *espW_1–206_* were amplified using primers P10/P12 with plasmids pICC1727 and pICC1728, respectively, and were cloned into the EcoRI/BamHI restriction sites of pGBT9 (Clontech), generating plasmids pICC1714 and pICC1715. Kif15_1092–1368_ was identified as a binding partner for EspW by a yeast two-hybrid screen (Clontech). *kif15_1092–1368_* was amplified by PCR with primers P13/P14 and cloned into pGAD-T7-AD (Clontech) using NdeI/XhoI as restriction sites, generating plasmid pICC1723. We used plasmid pICC1723 as a template to amplify *kif15_1142–1347_*, *kif15_1092–1347_*, and *kif15_1142–1368_* with primers P15/P16, P17/P16, and P15/P18, respectively. The genes were cloned into pGAD-T7 using NdeI/XhoI restriction sites, generating plasmids pICC1724, pICC1725, and pICC1726. Plasmid pICC1752, containing *kif15_1092–1142_*, was generated by inverse PCR using plasmid pICC1723 as the template and phosphorylated primers P19/P20. *kif15_1092–1368_* was cloned into the EcoRI/HindIII sites of the bacterial protein expression vector pRK5-Myc (Clontech) following amplification using primer pair P21/P22 and plasmid pICC1723 as a template, generating plasmid pICC1914. EPEC clinical isolates were screened first for the presence of *espW* by PCR using primer pair P29/P30. We further screened all of the *espW*-negative strains for the presence of *espW_1–206_* using primer pair P31/P32.

### Yeast two-hybrid assays.

Yeast two-hybrid screening using EspW as prey and a cDNA library as bait was performed as described previously (43). Briefly, a pretransformed MATCHMAKER HeLa cell cDNA library (Clontech) was screened according to the manufacturer's protocol for proteins interacting with EspW. The lithium acetate method was used to transform pGBT9-espW (pICC1714) (Table 2) into yeast strain AH109 (MAT**a**), and transformants were selected on Trp-minus-synthetic-defined agar plates. Following mating with the Y187 (MATα) yeast strain containing the cDNA library, diploids cells were selected on DDO and QDO media for selection of protein interactions. The cDNA-containing pGADT7 plasmid was rescued from positive clones and the cDNA identified by DNA sequencing. The prey plasmid and derivatives (Table 2) were then retransformed into AH109 either on its own to determine possible self-activation or with pICC1714 or pICC1715 to confirm interaction by direct yeast two-hybrid assay.

### Infection of Swiss 3T3 and HeLa cells.

Forty-eight hours prior to infection, Swiss 3T3 or HeLa cells were seeded in 24-well plates containing 13-mm glass coverslips (VWR International) at a density of 5 × 10^5^ cells per well. Before infection, the cells were washed 3 times with phosphate-buffered saline (PBS) and the medium was replaced with fresh DMEM without FCS. Cells in 24-well plates were infected with 20 μl of primed cultures. The plates were then centrifuged at 200 rpm for 5 min at room temperature, and infections were carried out for 3 h at 37°C in 5% CO_2_ without agitation. After infection, monolayers were washed at least 10 times in PBS to remove the bacteria and were fixed for immunofluorescence (to assess cell morphology) as described below.

For sphingosine 1-phosphate (S1P) (Sigma-Aldrich) treatment, S1P was dissolved in dimethyl sulfoxide (DMSO) (Sigma-Aldrich) and added to DMEM to attain a final concentration of 100 nM.

### Transfection.

Swiss 3T3 and HeLa cells were transfected for 24 h using Lipofectamine 2000 (Invitrogen) and GeneJuice (Merck Millipore), respectively, according to the manufacturer's instructions.

### Immunofluorescence and microscopy.

Coverslips were fixed with 3% paraformaldehyde (PFA) for 15 min before washing 3 more times with PBS. Cells were quenched for 10 min with 50 mM NH_4_Cl, permeabilized for 4 min in PBS–0.2% Triton X-100, and washed 3 times in PBS. The coverslips were blocked for 15 min in 0.2% bovine serum albumin (BSA)–PBS before incubation with primary and secondary antibodies. The primary antibodies mouse anti-hemagglutinin (HA) (Cambridge Bioscience), chicken anti-Myc (Millipore), and rabbit polyclonal anti-O157 (Roberto la Ragione, Veterinary Laboratory Agency, United Kingdom) were used at a dilution of 1:500. Coverslips were incubated with the primary antibody for 1 h, washed 3 times in PBS, and incubated with the secondary antibodies. AMCA-, Cy2-, RRX-, or Cy5-conjugated donkey anti-mouse, anti-chicken, and anti-rabbit antibodies (Jackson ImmunoResearch) were used as secondary antibodies. All dilutions were in PBS–0.2% BSA. Actin was detected with tetramethyl rhodamine isothiocyanate (TRITC)-conjugated phalloidin (1:500 dilution) (Sigma), phalloidin Alexa Fluor 350, or phalloidin Oregon green 488 (1:100 dilution) (Invitrogen). DNA was stained with 4′,6-diamidino-2-phenylindole (DAPI) (1:1,000 dilution). Coverslips were mounted on slides using ProLong Gold antifade reagent (Invitrogen) and examined by conventional epifluorescence microscopy using a Zeiss Axio LSM-510 microscope. Images were deconvoluted, processed using Axio Vision 4.8 LE software (Zeiss), and trimmed using Adobe Photoshop CS4.

### SEM.

For scanning electron microscopy (SEM), cells were washed 3 times in phosphate buffer, pH 7.4, and then fixed with 2.5% glutaraldehyde in phosphate buffer, pH 7.4. Cells were washed 3 times in phosphate buffer before being postfixed in 1% osmium tetroxide for 1 h. Cells were then washed 3 times in phosphate buffer and dehydrated for 15 min in graded ethanol solutions from 50% to 100%. The cells were then transferred to an Emitech K850 critical point drier and processed according to the manufacturer's instructions. The coverslips were coated in gold/palladium mix using an Emitech Sc762 mini sputter. Samples for SEM were then examined blindly at an accelerating voltage of 25 kV using a JEOL JSM-6390.

### Statistical analysis.

All data were analyzed with GraphPad Prism software, using one-way analysis of variance (ANOVA). Results were expressed as means and standard deviations (SD). Statistical significance was determined by a two-tailed Student *t* test. A *P* value of <0.05 was considered significant.

## Supplementary Material

Supplemental material
