# Supplementary material for: The Enterohemorrhagic Escherichia coli Effector EspW Triggers Actin Remodeling in a Rac1-Dependent Manner
Source: Infect Immun. 2017 Aug 18;85(9):e00244-17. doi: 10.1128/IAI.00244-17 (PMC5563575; doi:10.1128/IAI.00244-17)
Supplement: Supplemental material [file supp_85_9_e00244-17__index.html]

The Enterohemorrhagic Escherichia coli Effector EspW Triggers Actin Remodeling in a Rac1-Dependent Manner — Supplemental material 

# The Enterohemorrhagic Escherichia coli Effector EspW Triggers Actin Remodeling in a Rac1-Dependent Manner

## Supplemental material

- Supplemental file 1 -

  Table S1. List of primers. Fig. S1. Gene organization and protein identity. Fig. S2. Gene alignment. Fig. S3. Gene alignment.

  PDF, 1.1M
