# Supplementary material for: The Enterohemorrhagic Escherichia coli Effector EspW Triggers Actin Remodeling in a Rac1-Dependent Manner
Source: Infect Immun. 2017 Aug 18;85(9):e00244-17. doi: 10.1128/IAI.00244-17 (PMC5563575; doi:10.1128/IAI.00244-17)
Supplement: Supplemental material [file IAI.00244-17_zii999092130s1.pdf]

**Figure S1: Gene organisation and protein identity**

Genetic organisation of the SP17 pathogenicity island (**A**) and percentage identity of the EspW effectors (**B**) in different strains.

**Figure S2: Gene alignment**

Multi-alignment of *espW* genes from EHEC O157:H7, O111:H-, O26:H11, O103:H2 and O55:H7.

**Figure S3: Gene alignment**

Following infection of transfected Kif151092-1368 (green) cells, Kif151092-1368 localized at the actin (red) pedestals (white arrows), under adherent EHEC Δ*espW* (magenta). DNA was visualized by Hoechst staining (blue).

**Table S1.** List of primers

| Names | Primer sequence (5'-3') | Restriction enzyme |
|---|---|---|
| P1 | CGCGGATCCATGCCCAAAATATCATCAGTTGTATC | BamHI |
| P2 | CCGGAATTCTTAATTTCTAACCAAGGGGTCCC | EcoRI |
| P3 | CCGGAATTCTCAATCAGAACTAATATTTTCGAACATTCTTACG | EcoRI |
| P4 | TCGGATCCGTGAGCAAGGGCGAGGAG | BamHI |
| P5 | CAGAATTCCTTGTACAGCTCGTCCATGC | EcoRI |
| P10 | CCGGAATTCATGCCCAAAATATCATCAGTTGTATC | EcoRI |
| P11 | TGCACTGCAGTTAATTTCTAACCAAGGGGTCCC | PstI |
| P12 | CCGGGATCCTTAATTTCTAACCAAGGGGTCCC | BamHI |
| P13 | CTATTCGATGATGAAGATACCCCACCAAACCC | - |
| P14 | GTGAACTTGCGGGGTTTTTCAGTATCTACGAT | - |
| P15 | CGCCATATGatgAAGACACCACCTCACTTTCAAAC | NdeI |
| P16 | CGGTCGATTGGTGACCTACCAACTTTCCA | XhoI |
| P17 | CGCCATATGATGGAACTGACCAAGAAGGAAGCC | NdeI |
| P18 | CCGCTCGAGCTCAGCAAGCCTGACATTTTCC | XhoI |
| P19 | TCGAGCTGCAGATGAATCGTAG | - |
| P20 | GAGGACTCTGGGGATCCTC | - |
| P21 | CCGGAATTCcATGGAACTGACCAAGAAGGAAGCC | EcoRI |
| P22 | CCCAAGCTTTTACTCAGCAAGCCTGACATTTTCC | HindIII |
| P23 | TTTCCTGTACTGAGGAACATC | - |
| P24 | CCGATAACGCTGGACACAC | - |
| P25 | CCGGAATTCGGGCATAAATAGTCTCCTCG | EcoRI |
| P26 | CCGGAATTCCATGGGACCCCTTGGTTAG | EcoRI |
| P27 | AGACAACTTAACGGAGGTAAC | - |
| P28 | TCAGAGTGTCATGGCAGAAC | - |
| P29 | AATATCATCAGTTGTATCATCATG | - |
| P30 | AGCGTTTATCATCACTGTTTATG | - |
| P31 | ATGGTTGGCTACAGCTAAAACTC | - |
| P32 | CCAGAAGAACAATTACCTTGTG | - |

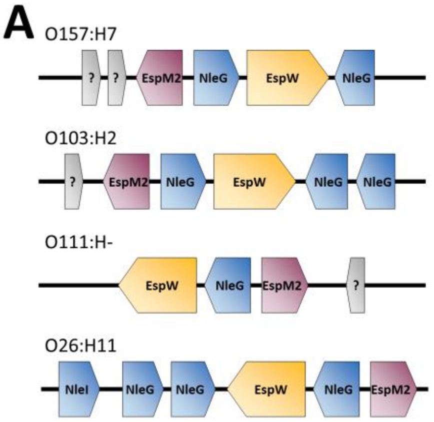


**B**

| | O157:H7 | O111:H- | *Shigella boydii* | O26:H11 | O111:H11 | O103:H25 | O103:H2 |
|---|---|---|---|---|---|---|---|
| O157:H7 | 100.0% | | | | | | |
| O111:H- | 99.7% | 100.0% | | | | | |
| *Shigella boydii* | 99.4% | 99.7% | 100.0% | | | | |
| O26:H11 | 98.3% | 98.3% | 98.6% | 100.0% | | | |
| O111:H11 | 98.3% | 98.3% | 98.6% | 100.0% | 100.0% | | |
| O103:H25 | 98.3% | 98.3% | 98.6% | 100.0% | 100.0% | 100.0% | |
| O103:H2 | 94.3% | 94.6% | 97.2% | 96.3% | 96.3% | 96.3% | 100.0% |

Fig. S1

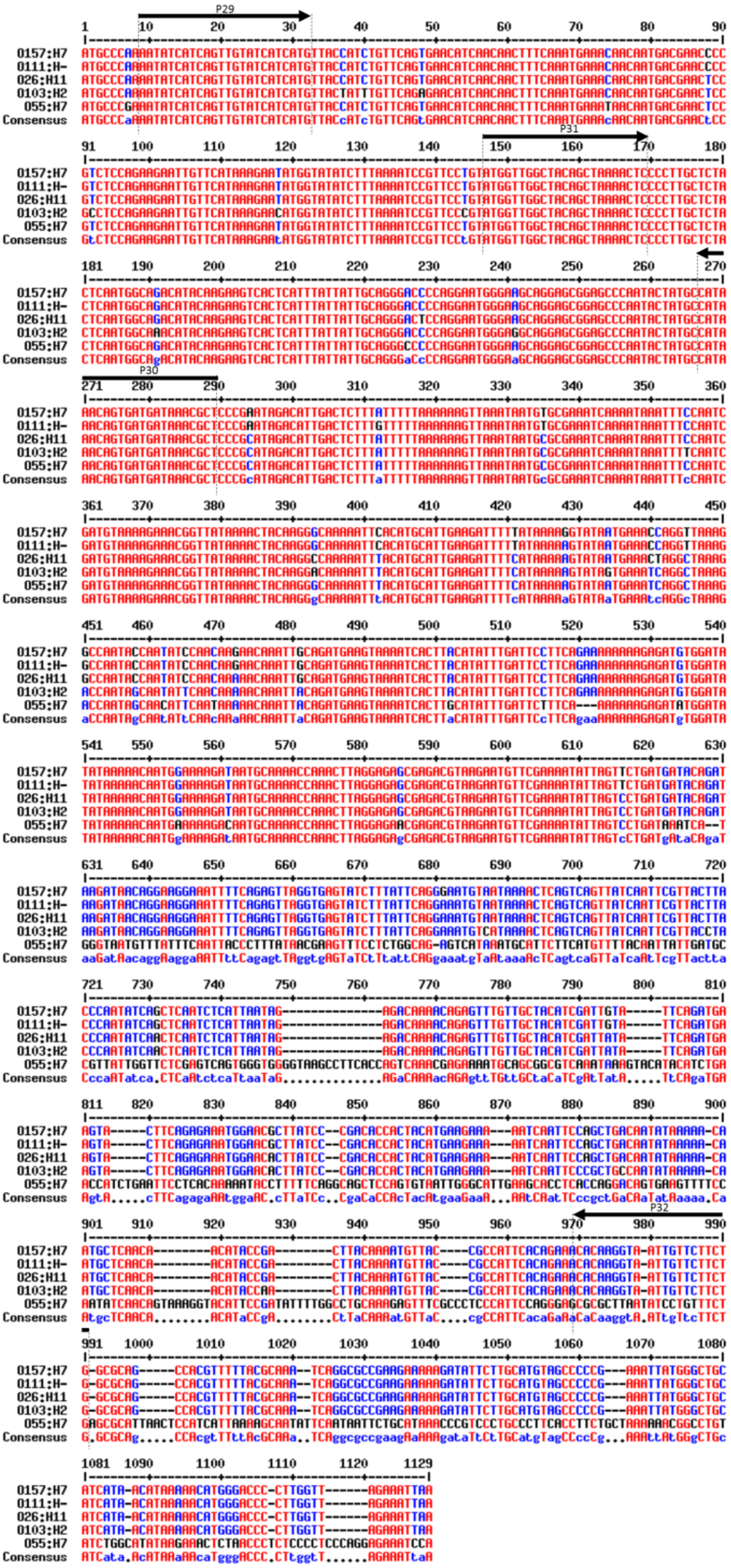


Fig. S2

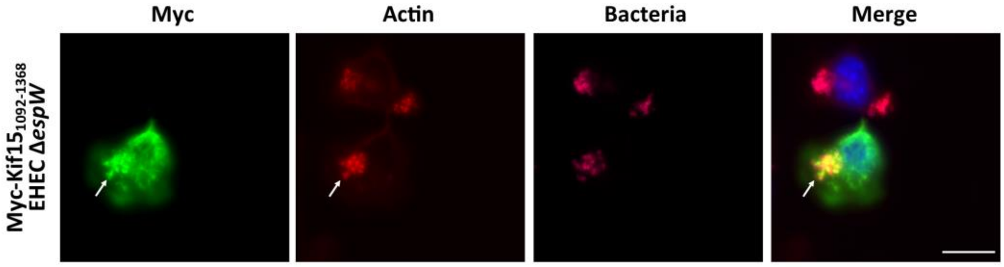


Fig. S3
